# All-Perfluorosulfonated-Ionomer Composite Membranes Containing Blow-Spun Fibers: Effect of a Thin Fiber Framework on Proton Conductivity and Mechanical Properties

**DOI:** 10.1021/acsami.3c17643

**Published:** 2024-02-21

**Authors:** Shuta Onuki, Yoshiki Kawai, Hiroyasu Masunaga, Noboru Ohta, Ryohei Kikuchi, Minoru Ashizawa, Yuta Nabae, Hidetoshi Matsumoto

**Affiliations:** † Department of Materials Science and Engineering, Tokyo Institute of Technology, 2-12-1 Ookayama, Meguro-ku, Tokyo 152-8552, Japan; ‡ Japan Synchrotron Radiation Research Institute, 1-1-1 Kouto, Sayo, Hyogo 679-5198, Japan; § Materials Analysis Division, Open Facility Center, Tokyo Institute of Technology, 2-12-1 Ookayama, Meguro-ku, Tokyo 152-8550, Japan

**Keywords:** perfluorosulfonated ionomer, thin fiber, composite, membrane, fuel cell

## Abstract

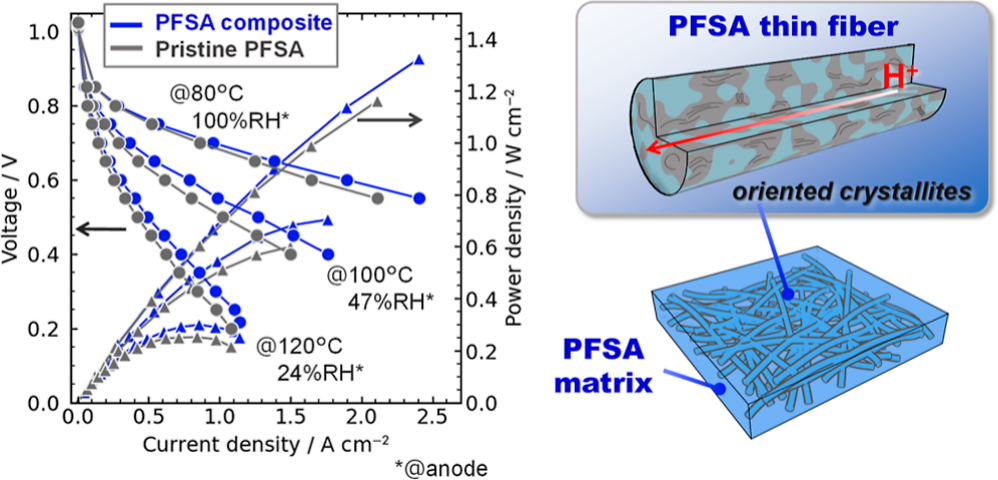

In this study, thin fiber composite polymer electrolyte membranes (PEMs) were prepared using short side-chain perfluorosulfonic acid (PFSA) ionomers, Aquivion, to create composite PEMs with improved proton conductivity and improved mechanical properties. PFSA thin fiber webs prepared by blow spinning and successive hot pressing were used as the porous substrate. Herein, PFSA ionomers were used for both the substrate and the matrix of the composite PEMs, and their structures, properties, and fuel cell performance were characterized. By adding the PFSA thin fiber webs to the matrix, the proton conductivity was enhanced and the mechanical properties were slightly improved. The prepared PFSA thin fiber composite PEM showed better FC performance than that of the pristine PFSA one for the high-temperature low-humidity condition in addition to the low-temperature high-humidity one. To the best of our knowledge, this is the first report on the all PFSA composite membranes containing a PFSA thin fiber framework.

## Introduction

Recently, polymer electrolyte fuel cells (PEFCs) have become the most attractive electrochemical power converter because of their wide variety of applications, such as in automotive power, stationary power, and microelectronics.^1,2^ PEFCs convert the chemical energy of hydrogen and oxygen fuels directly into electricity, affording devices with a high power density, zero CO_2_ emissions, and low operating temperatures. Polymer electrolyte membranes (PEMs) are a crucial component of PEFCs, determining their performance and durability.^3^

Perfluorosulfonated (PFSA) ionomers, a random copolymer consisting of semicrystalline polytetrafluoroethylene (PTFE) backbone and pendant side chains terminated by sulfonic acid groups, are commonly used as PEMs for PEFCs because of their high proton conductivity and excellent chemical stability.^4^ Conventional PEMs that are used in commercial PEFCs are the PFSA ionomer membranes reinforced with porous expanded PTFE (such as Gore-Select and Nafion XL).^2,4,5^ Recently, highly porous electrospun poly(vinylidene fluoride) (PVDF) nanofibers were used as the reinforcement to improve the dimensional stability of PFSA ionomer membranes.^6,7^ In addition, ion-conductive nanofibers can increase ionic transport pathways in a polymer matrix compared with ion-insulating nanofibers such as PVDF nanofibers.^8^ In line with this scenario, there are many reports on preparation of composite PEMs containing ion-conductive nanofibers for the electrochemical devices including fuel cells.^9−12^ The ion-conductive three-dimensional (3D) nanofiber framework can improve the electrochemical properties of the composites and mechanically reinforce the polymer matrix.^13−15^

To improve proton conductivity through the composite PFSA PEMs, we fabricated PFSA thin fibers with an average diameter of 1.2 μm and used them as a highly porous substrate with proton conductivity. PFSA thin fibers were prepared aerodynamically by blow spinning from PFSA aqueous dispersion^16,17^ because inherent electrical charges of PFSA dispersions (i.e., dissociated sulfonic acid groups) can prevent charging and/or cause instability of the polymer jet under a high electric field for the commonly used electrospinning.^18^ Herein, a representative PFSA ionomer with a short-side chain, Aquivion,^19^ was used as both a polymer matrix and a thin fiber framework. Some researchers reported the preparation of PFSA ionomer nanomicro scaled thin fibers including Aquivion.^20^ However, studies on PFSA composite electrolyte membranes containing PFSA thin fibers have not been reported.^18,21^ In this work, we prepared all PFSA composite electrolyte membranes containing a well-interconnected PFSA thin fiber framework. To enhance the stability of the Aquivion ionomer and increase the fiber connectivity, we carried out the successive hot pressing of the as-spun PFSA thin fiber framework prepared by blow spinning and then used it as a stable proton-conductive reinforcement. This work aims to examine the effect of the PFSA thin fiber framework on the electrochemical and mechanical properties and initial PEFC performances of all the PFSA composite electrolyte membranes containing thin fibers.

## Experimental Section

### Materials

A 25 wt % Aquivion aqueous dispersion (D72-25BS, equivalent weight of 720 g equiv^–1^) was purchased from Solvay, Belgium. Poly(ethylene oxide) (PEO, *M*_w_ = 4,000,000 g mol^–1^) and poly(vinylidene fluoride) (PVDF, *M*_w_ = 275,000 g mol^–1^) were purchased from Polysciences Inc., United States and Sigma-Aldrich, United States, respectively. *N*,*N*-Dimethylacetamide (DMAc; purity, ≥98.0%), ethanol (EtOH; purity, ≥99.5%), potassium chloride (KCl; purity, ≥99.0%), 1 mol L^–1^ (M) hydrochloric acid (HCl; for volumetric analysis), and 0.01 M potassium hydroxide (KOH; for volumetric analysis) were purchased from Fujifilm Wako, Japan. These reagents were used as received without further purification. Ultrapure water was prepared by using a water purification system (Milli-Q Advantage, Merck Millipore, Germany) and then used as an aqueous solution.

### Blow Spinning

PEO (as a spinning aid) aqueous solution was added to a 25 wt % Aquivion aqueous dispersion, and the mixture was stirred at 25 °C for 24 h to obtain a spinning solution (the final composition was Aquivion/PEO/water = 12.5:0.625:86.875 by weight). The blow spinning device was the same as that used in our previous study (Figure S1, Supporting Information).^17^ The spinning solution was contained in a syringe with a stainless-steel nozzle (0.14 mm internal diameter). A constant volume flow rate of 0.36 mL h^–1^ was maintained using a syringe pump (KDS100, KD Scientific Co., USA). Compressed dry air (air pressure of approximately 0.02–0.04 MPa) was delivered to the nozzle via an oil-free scroll compressor (SLP-15EFDM5, ANEST IWATA Corporation, Japan). Wire netting was used as the collector. The nozzle-to-collector distance was 400 mm. An IR lamp (100 W, Vivaria, Japan) was placed near the nozzle tip to promote solvent evaporation. To produce aligned thin fibers, two Cu pipes (diameter: 2 mm) were placed parallel to each other and used as the collector similarly as that used in our previous study.^17^ The as-spun PFSA thin fiber webs were annealed at 190 °C for 12 min and 210 °C for 6 min according to the annealing procedure by a manufacturer.^23^ The annealed PFSA thin fiber webs were hot-pressed using a press apparatus (H300-01, As One, Japan) at 170 °C (the temperature is between the glass transition point of ∼120 °C and melting point of ∼185 °C^22^) under the pressure of 0.35 MPa for 10 min. The hot-pressing was carried out in order to enhance the crystallinity of Aquivion and weld the cross-points of the fibers in the webs. For comparison, ion-insulating thin fiber webs were prepared by electrospinning from a 35 wt % PVDF/DMAc solution and hot-pressed at 140 °C under the pressure of 1.0 MPa for 10 min.

### Membrane Preparation

Thin fiber composite membranes were prepared by a casting method. A 25 wt % Aquivion aqueous dispersion was cast on the hot-pressed PFSA thin fiber webs and PVDF thin fiber webs. The casted membranes were dried at 25 °C for 1 h, at 80 °C for 30 min, and annealed at 190 °C for 12 min and 210 °C for 6 min according to the annealing procedure by a manufacturer.^23^ Before use, all the prepared PFSA thin fiber composite membranes were immersed in a 1 M HCl aqueous solution for 24 h to remove PEO (spinning aid) from the PFSA thin fiber, were then washed sufficiently with ultrapure water, and vacuum-dried at room temperature. For comparison, we prepared the annealed PFSA membrane without containing thin fiber webs, which was named the “pristine PFSA membrane”. The membrane thickness was determined by the height gauge (Digimatic indicator ID-C112AXB, Mitutoyo, Japan).

### Characterization

The morphologies of the prepared thin fiber webs and membranes were observed by scanning electron microscopy (SEM, JCM-5700, JEOL, Japan) operated at 5 kV and field-emission scanning electron microscopy (FE-SEM, S4700, Hitachi High-Tech Corporation, Japan) operated at 3 kV. The samples were sputter-coated with Pt and osmium for SEM and FE-SEM observations, respectively. The fiber diameter distribution was determined by SEM image analysis using ImageJ software (National Institutes of Health, Bethesda, MD, USA). The internal structure of the single PFSA thin fiber was observed by transmission electron microscopy (TEM, H7650 Zero A, Hitachi High-Tech Corporation, Japan) operated at 100 kV. The aligned fiber samples were immersed in a 1 M aqueous AgNO_3_ solution for 12 h to ensure that the counterions were exchanged with Ag^+^. Then, the samples were washed sufficiently with ultrapure water and vacuum-dried at room temperature. Thereafter, the fibers were embedded into UV-curable resin and sectioned to a thickness of ∼80 nm by using an ultramicrotome (Leica UC7, Leica Microsystems GmbH, Germany). The ultrathin sections were transferred to a colloidal-coated copper grid.

Small-angle X-ray scattering (SAXS) measurements were performed at BL40B2 in the SPring-8 synchrotron radiation facility (Hyogo, Japan). The aligned thin fibers and the prepared membranes were irradiated with X-rays of wavelength (λ) = 0.06 and 0.1 nm. The scattering patterns were recorded on a PILATUS 3S 2 M detector (Dectris, Switzerland) located 1.06 and 2.17 m from the sample. The Fourier transform infrared (FTIR) spectra of the as-spun thin fibers and the prepared composite membranes after acid treatment were recorded by using an FTIR spectrometer (FT/IR-6300, JASCO, Japan) with an attenuated total reflection unit (PRO670H-S, JASCO, Japan), including a diamond crystal. Thermogravimetric analysis (TGA) curves of the prepared composite and pristine PFSA membranes were measured using a Rigaku Thermo plus EVO TG 8120 thermal analyzer under an N_2_ atmosphere, by heating from 25 to 350 °C at a rate of 10 °C min^–1^. The differential scanning calorimetry (DSC) measurements of the prepared composite and pristine PFSA membranes were performed by using a DSC7000X calorimeter (Hitachi High-Tech Corporation, Japan) under a N_2_ atmosphere by heating from 50 to 220 °C at a rate of 1 °C min^–1^.

Potentiometric titration measurements were performed using a potentiometric titrator (888 Titrando, Metrohm, Switzerland). The prepared membranes were immersed in a 1 M aqueous HCl solution for 24 h to ensure that the counterions were saturatedly exchanged with H^+^.^24^ They were then washed sufficiently with ultrapure water to remove excess HCl (the electrical conductivity of the rinsing solution was almost the same as that of ultrapure water). Thereafter, the samples were soaked in a 1 M KCl solution for 24 h to elute H^+^. Then, the eluents were titrated by adding 0.01 M KOH solution to obtain the titration curves. The amount of fixed charge groups (*N*_*x*_) in the samples is equal to the titer of KOH. The ion-exchange capacity (IEC) was determined using the equation^10^
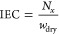
1where *w*_dry_ is the weight of the sample in the dry state. The samples were vacuum-dried at 25 °C for 20 h, and the dry weight was determined.

The water content of the membranes (*w*_w_) was calculated using as^24^
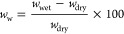
2where *w*_wet_ is the weight of the sample in the equilibrium swollen state. The samples were kept at 25 °C and 95% RH for 3 h in a constant temperature–humidity chamber (LHU-113, ESPEC Corp., Japan), and the swollen weight was measured.

The through-plane ionic conductivity of all of the membranes were measured using the alternating current (AC) impedance method with a potentio-galvanostat (SP-150, Bio-Logic, France) in the range of 0.1 Hz to 1 MHz. Symmetrical two-electrode cells made of Pt were used. The measurements were carried out at 20 °C/30% RH, 20 °C/65% RH, and 20 °C/85% RH conditions in a constant temperature–humidity chamber. The through-plane proton conductivity (σ [S/cm]) was calculated by the following equation
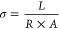
3where *L* is thickness of the membrane, *A* is the membrane area, and *R* is the membrane resistance.

The activation energy (*E*_a_) of the proton conductivity through the membranes was calculated using the following Arrhenius equation
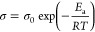
4where σ_0_ is the value of pre-exponential factor [S/cm], *E*_a_ is the activation energy required for protons to transport, *R* is the gas constant [J mol^–1^ K^–1^], and *T* is the absolute temperature [K].

Tensile tests of the aligned NF sheets were performed using a tensile tester (STA-1150, A&D, Japan). The test samples were cut to a width of 5 mm and a length of 15 mm. All measurements were performed at an elongation rate of 3 mm min^–1^ at room temperature. Five samples were measured for each sheet, and the mean value (±standard error) was calculated except PVDF thin fiber composite membrane.

### Single-Cell Fuel Cell Fabrication and Performance Evaluation

A carbon-supported Pt-catalyst loaded gas diffusion layer (GDL, 28 BC, SGL, 0.5 mg of Pt cm^–2^) with a microporous layer was purchased from Chemix, Japan, and used as-received. Membrane electrode assemblies (MEAs) were prepared by pressing the two GDLs (1 cm^2^ area) and a membrane prepared at 150 °C and 2 MPa for 2 min. The MEA was set in a test cell (1 cm^2^ area, FC-004, Chemix, Japan) and was evaluated using AutoPEM (Toyo Corporation, Japan). Before testing, the initial conditioning of the cells was carried out for 1 h. The *I*–*V* performance was measured under three conditions: 80 °C (100% RH@anode and 81% RH@cathode), 100 °C (47% RH@anode and 38% RH@cathode), and 120 °C (24% RH@anode and 19% RH@cathode). The humidified H_2_ and O_2_ were supplied into the anode and the cathode, respectively, at a flow rate of 200 mL min^–1^ without back pressure. The polarization curves were measured by recording the current density after holding the cell voltage with an electronic load unit (890e, Scribner, USA) for 5 min at each value. The electrochemical impedance spectroscopy (EIS) measurements were performed by applying AC amplitude corresponding to 10% of the direct current in the frequency range of 10 kHz to 0.1 Hz using the same electronic load.

## Results and Discussion

### Characterization of the PFSA Thin Fibers

**Figure 1:**
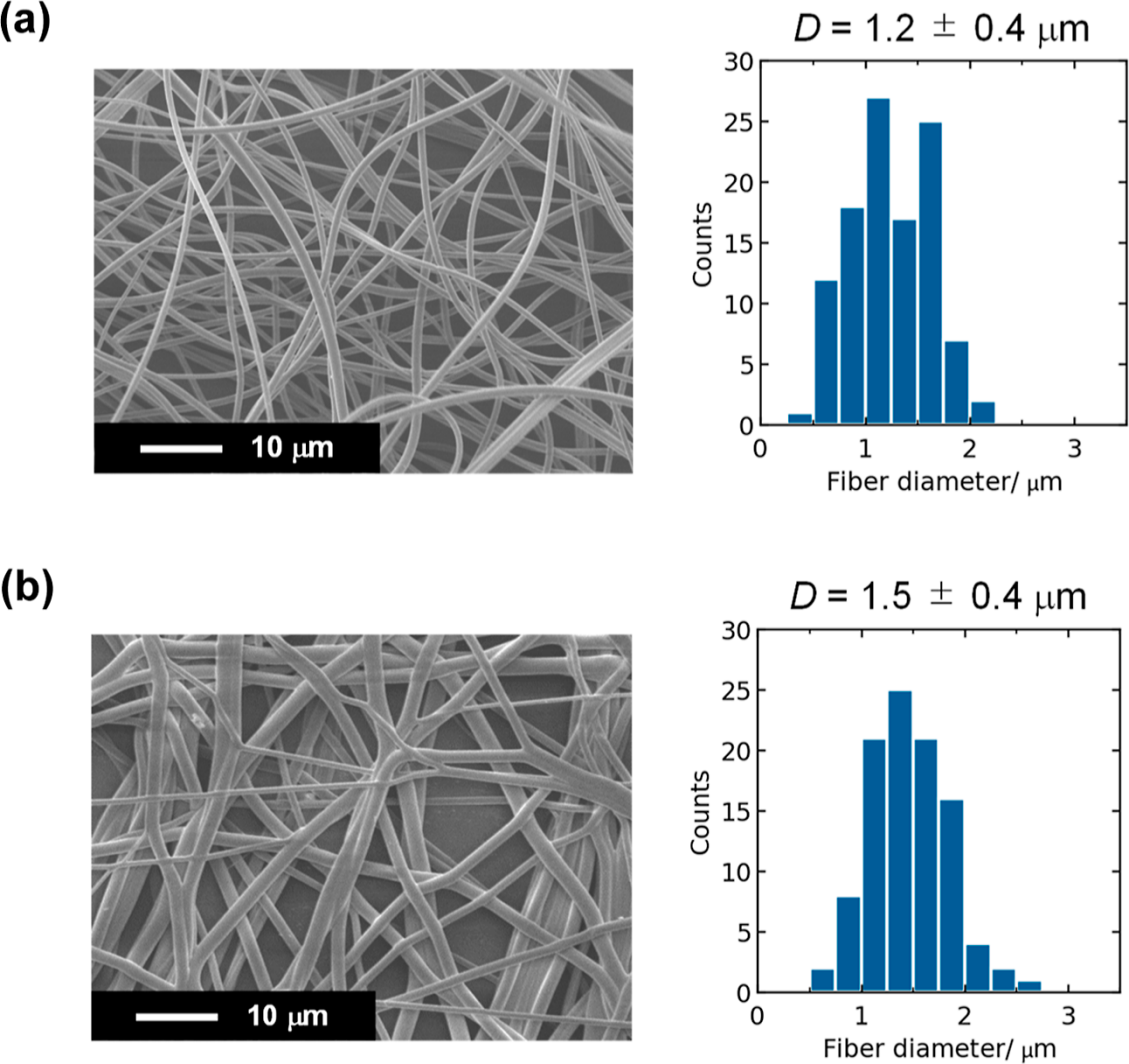
Surface SEM images and their fiber diameter distributions determined by SEM image analysis for the (a) as-spun and (b) hot-pressed PFSA thin fiber webs.

Figure 1 shows the surface SEM images and fiber diameter distribution for the as-spun and hot-pressed PFSA thin fibers. After hot-pressing, the cross-points of the fibers were partially welded, and the average fiber diameter increased to 1.5 ± 0.4 from 1.2 ± 0.4 μm. For comparison, the hot-pressed PVDF thin fiber web with the average diameter of 2.5 ± 0.6 μm was also prepared (the SEM is shown in Figure S2, Supporting Information). The hot-pressed PFSA and PVDF thin fiber webs were used as the porous substrates for the preparation of thin fiber composite PEMs.

**Figure 2:**
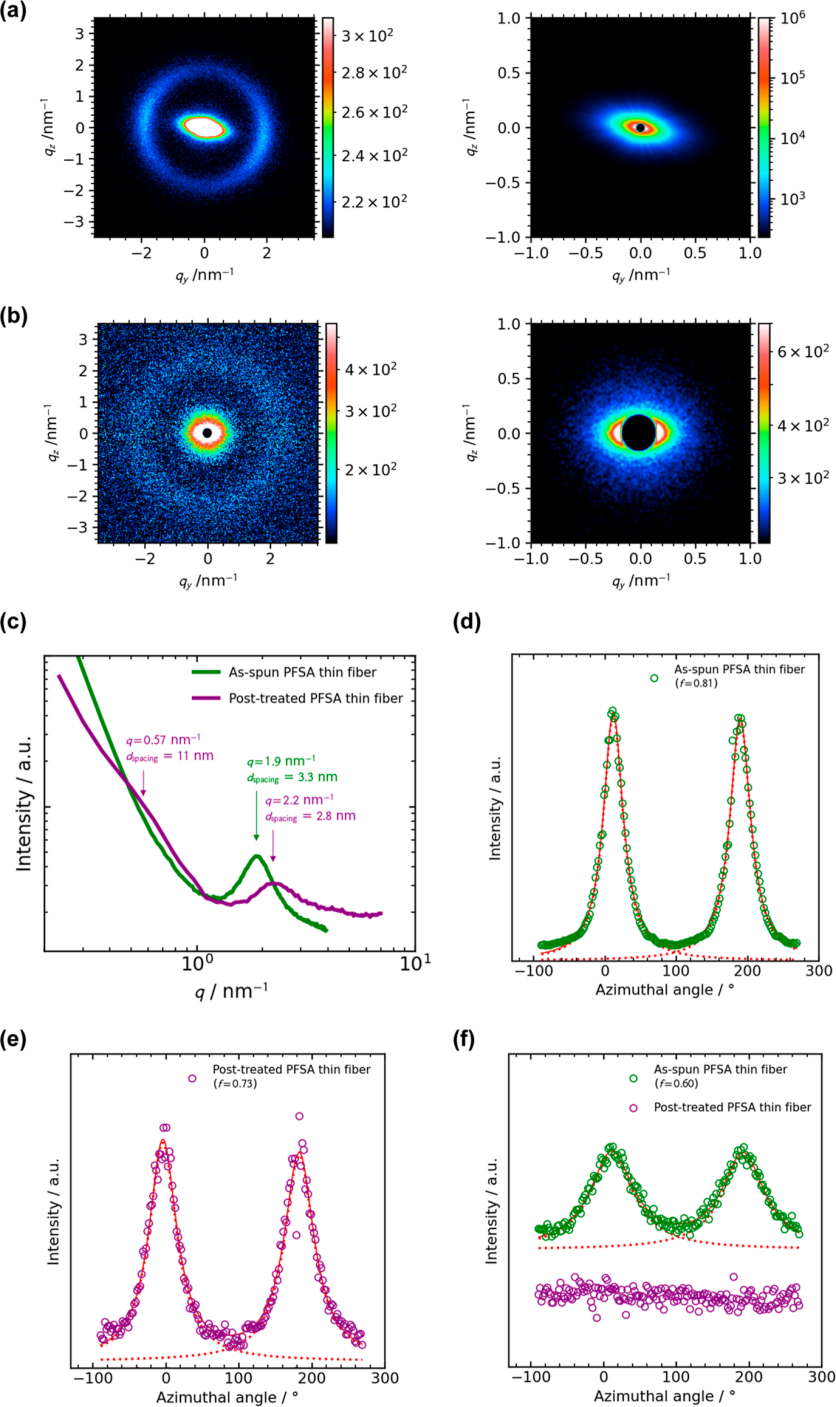
2D SAXS patterns of (a) the as-spun and (b) post-treated aligned PFSA thin fibers. Right-side images are the enlarged patterns of the left-side ones. The meridian direction of (a,b) is the fiber axis direction. (c) 1D profiles of (a,b). The azimuthal profiles of the matrix-knee at *q* = 0–1 nm^–1^ of the (d) as-spun and (f) post-treated aligned PFSA thin fibers. (e) The azimuthal profiles of the ionomer peak at *q* = 2–3 nm^–1^ of the as-spun and post-treated aligned PFSA thin fibers. All SAXS measurements were carried out at 25 ± 1 °C and 30–40% RH.

To clarify the internal structures of the PFSA thin fibers prepared by blow spinning, SAXS measurements were performed for the aligned PFSA thin fibers (the second-order parameter *S* of 0.86, the SEM image is shown in Figure S3, Supporting Information). For comparison, we prepared the annealed, hot-pressed, and acid-treated aligned PFSA thin fibers as a model of the fibers inside the composite membranes, which was named “post-treated thin fibers”. The 2D SAXS patterns and 1D profiles of the as-spun and post-treated aligned PFSA thin fibers measured at 25 ± 1 °C and 30–40% RH are shown in Figure 2a–c. Both the enlarged 2D patterns were anisotropic, and the shoulder was observed at *q* = 0.57 nm^–1^ in the 1D profile of post-treated thin fibers (Figure 2c), corresponding to the so-called matrix knee (intercrystalline domain spacing of ∼11 nm). The peak was observed at *q* = 1.9–2.2 nm^–1^ in the 1D profile, corresponding to the so-called ionomer peak (center-to-center distance of the ionic domains of *d* = 3.3–2.8 nm for Aquivion),^25^ respectively. The azimuthal profiles of the matrix knee (Figure 2d,e) clearly indicate that the PTFE crystalline domains are oriented along the fiber axis direction in both the as-spun and post-treated fibers (orientation function *f* values are 0.81 and 0.71, respectively). On the other hand, ionic domains in the as-spun fibers are oriented along the fiber axis direction (Figure 2f, *f* = 0.6, similar orientation was reported for the electrospun PFSA nanofibers^26^), while the ionic domains in the post-treated fibers are isotropic. Typical TEM image of the PFSA thin fiber also support homogeneous distribution of the circular ionic domains in the fiber after post-treatment (Figure S4, Supporting Information, we could not exchange the counterion of the as-spun PFSA thin fiber with metal ions because Aquivion is water-soluble). The orientation of crystalline and ionic domains in the as-spun fibers would be ascribed to the higher extensional strain rate experienced by the individual fibers during blow spinning.^17^ In addition, the post treatment of the fibers including treatments at high temperatures (170–210 °C) slightly decreases the orientation of crystalline domains and greatly promotes structural relaxation of amorphous ionic domains. The ionomer peak also shifted to the higher *q* value by the post treatment. This shift is commonly observed for the annealed PFSA ionomers.^27^ One possibility is that microstructural change of the crystalline domains induced by the post treatment (we can confirm the clear shoulder at *q* = 0.57 nm^–1^ after the post-treatment in Figure 2c) decreases the center-to-center distance of the ionic domains.

### Thin Fiber Composite Membranes

Before use, acid treatment was carried out for all the prepared composite PFSA membranes to remove PEO. After acid treatment, the FT-IR spectra showed that the peaks at ∼2876 and ∼1466 cm^–1^, attributed to the C–H symmetric stretching and CH_2_ scissoring, respectively, disappeared,^28^ indicating that PEO was removed from the membrane (the FT-IR spectra are shown in Figure S5, Supporting Information). In addition, we investigated the effect of acid treatment on the morphology and internal structure of thin fibers. The morphology and fiber diameter distribution for the PFSA thin fiber webs before and after acid treatment is shown in Figures 1b and S6, respectively. After acid treatment, the average fiber diameter did not change. The TEM image of the single fiber after acid treatment also shows no indication of defect inside fiber (Figure S4).

**Figure 3:**
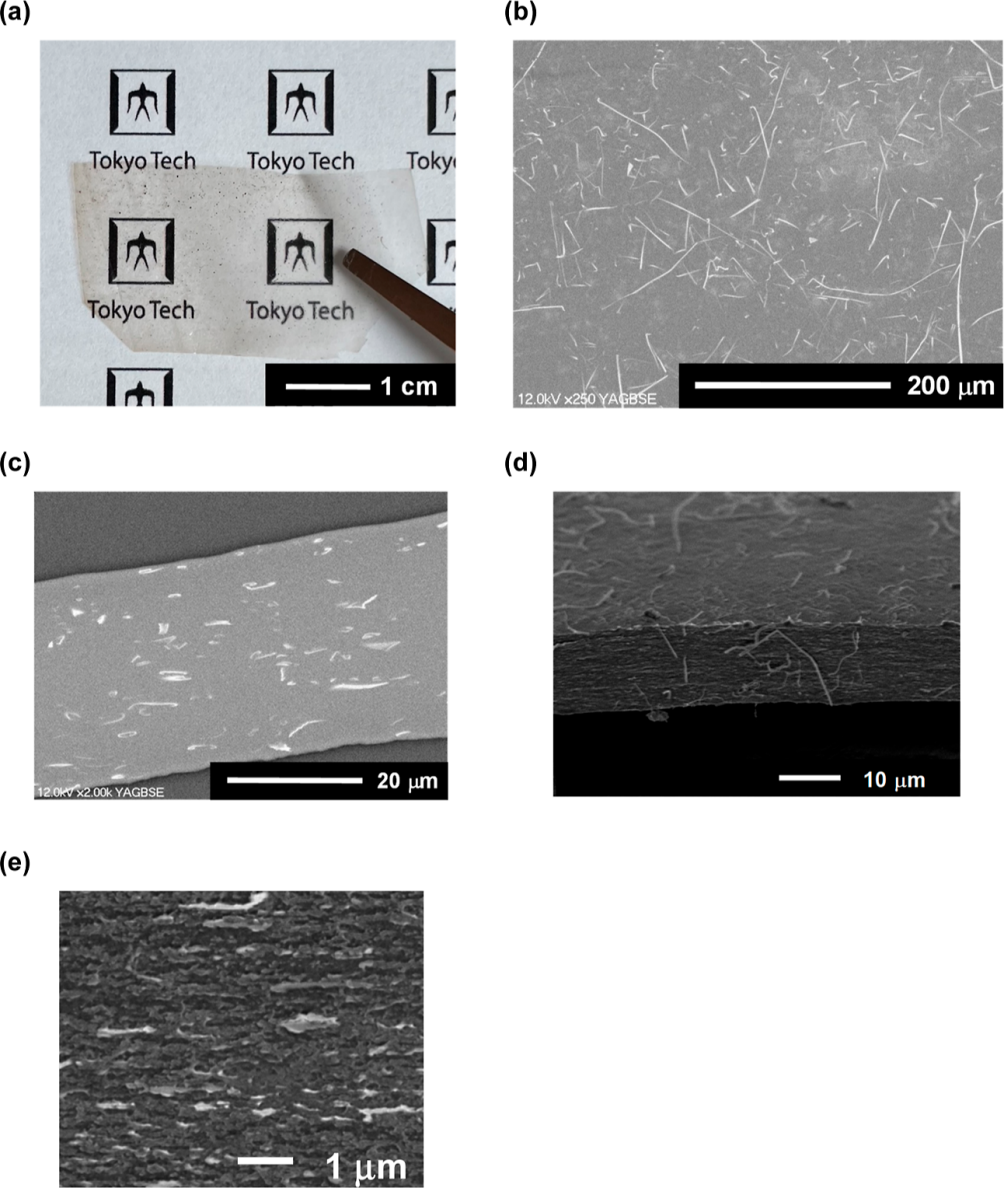
(a) Photograph and (b) surface and (c) cross-sectional FE-SEM images of composite PFSA membrane (thin fiber content of 15 wt %). (d) Cross-sectional SEM image of the fractured composite PFSA membrane (thin fiber content of 15 wt %) after tensile test. (e) Enlarged FE-SEM image of (d).

**Figure 4:**
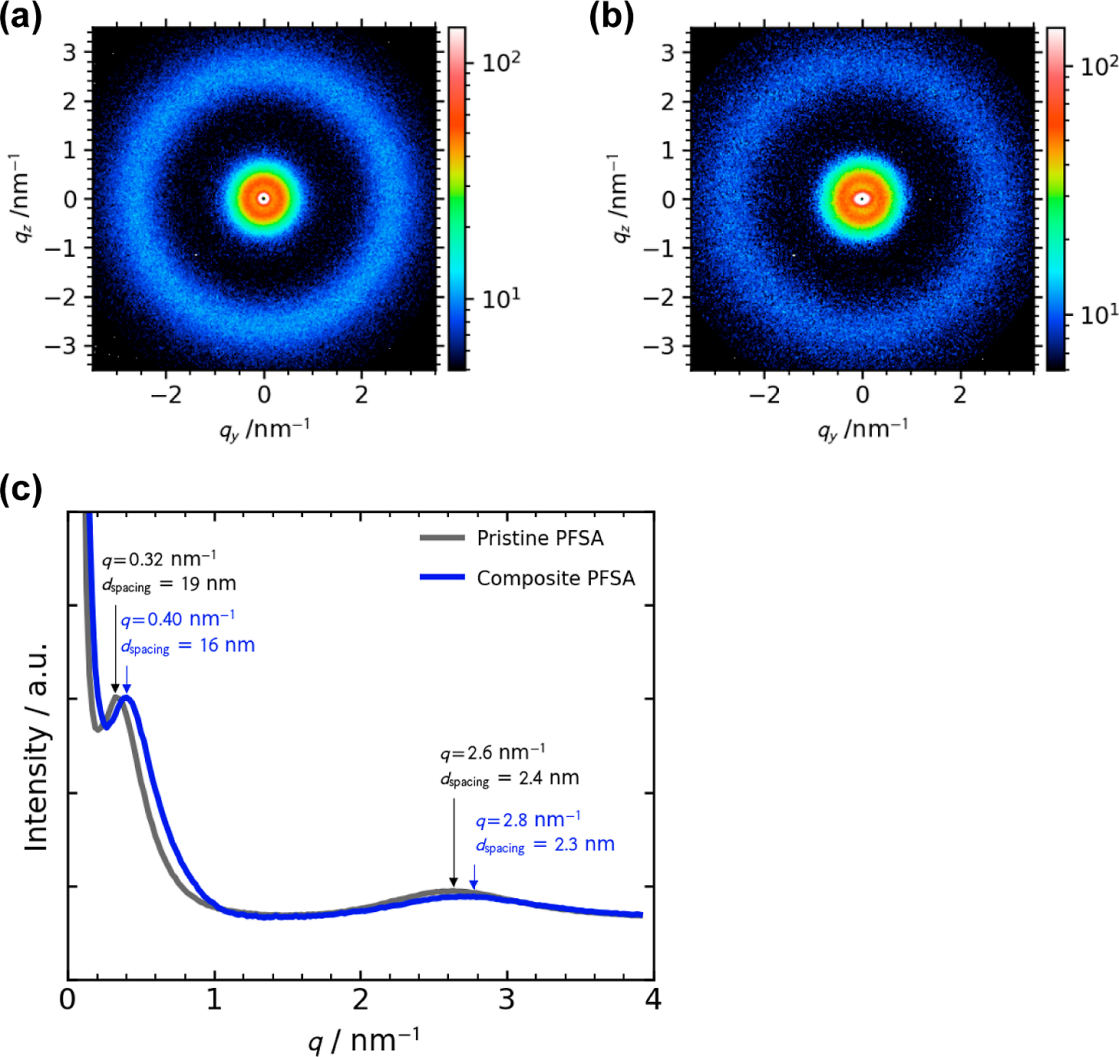
(a) Typical 2D SAXS patterns of the (a) pristine PFSA membrane and (b) composite PFSA membrane (thin fiber content of 15 wt %). (c) 1D profiles of (a,b). All SAXS measurements were carried out at 25 ± 1 °C and 20–30% RH.

Typical photographs and SEM images of the PFSA thin fiber composite membrane are shown in Figure 3. For Figure 3b,c, Pt-sputter-coated PFSA thin fiber webs were used in order to clearly distinguish the thin fiber and matrix. The prepared composite membrane was transparent (Figure 3a) and 35–40 μm thick (Figure 3c). For Figure 3d,e, the fractured sample after the tensile test was used in order to confirm the existence of defects inside the membrane (the stress–strain curve during tensile test is shown in Figure S7, Supporting Information) because the internal defects can act as the crack origin. These SEM images including a fracture surface showed that a defect-free dense microstructure containing well-dispersed thin fibers in a polymer matrix was formed. This microstructure is consistent with the transparency. Figure 4 shows the typical 2D SAXS patterns and 1D profiles of the prepared pristine and composite PFSA membranes measured at 25 ± 1 °C and 20–30% RH. The peaks were observed at *q* = 0.32–0.4 and 2.6–2.8 nm^–1^, corresponding to the distance of 16–19 nm between the crystalline domains of PTFE (so-called matrix knee^25^) and spacing of 2.3–2.4 nm between the ionic domains (aforementioned ionomer peak), respectively. These findings demonstrate that the addition of the thin fiber webs does not significantly change the microstructure of PFSA.

**Figure 5:**
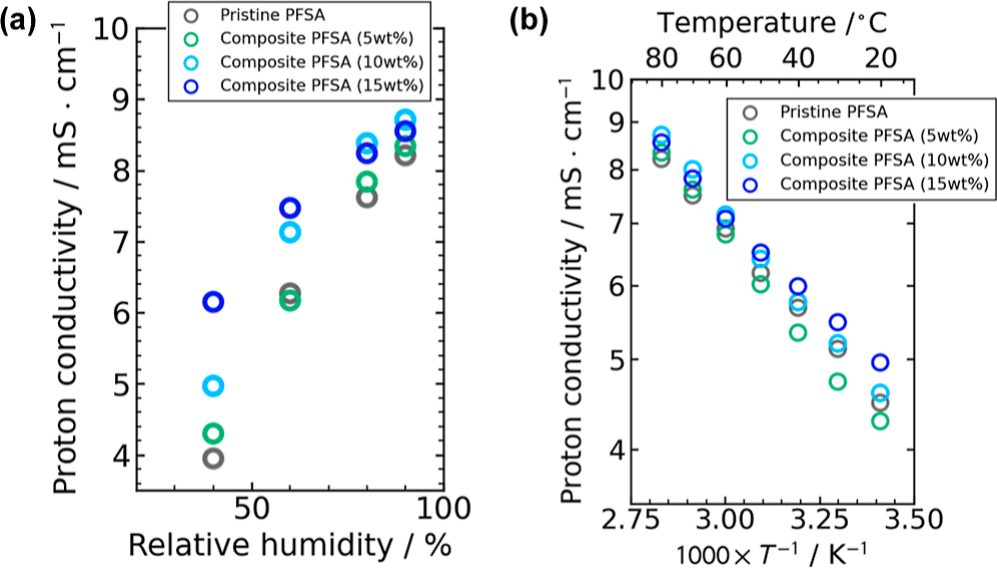
(a) Relative humidity dependence through the proton conductivity through the thin fiber composite and pristine PFSA membranes at 80 °C. (b) Temperature dependence of the proton conductivity through the thin fiber composite and pristine PFSA membranes at 90% RH.

The physicochemical properties of the prepared membranes are summarized in Table 1. All the prepared PFSA thin fiber composite membranes and pristine PFSA membrane showed a similar IEC of ∼1.3 mmol g^–1^, which corresponded to the calculated value of 1.39 mmol g^–1^ for the Aquivion with the EW of 720 g mol^–1^, and a similar water content of 35–36%. On the other hand, the composite membrane containing 15 wt % ion-insulating PVDF thin fibers showed a lower IEC of 1.0 mmol g^–1^ and water content of 27%. Compared to the pristine PFSA membrane and PFSA membranes containing PVDF thin fiber web, the proton conductivity through the PFSA thin fiber composite membranes were improved: the membrane with larger content of PFSA thin fiber showed a better conductivity. In particular, the additive effect of PFSA thin fibers was more substantial in the low humidity condition. The obtained proton conductivity values are smaller than the reported values for PFSA membranes (10–160 mS/cm^29−31^). Holdcroft et al. pointed out that both the configuration of measuring cell and measuring condition significantly influence the obtained proton conductivity by EIS measurements.^30^ Herein, we think that comparison of the obtained conductivities for the prepared membranes measured by the same cell and same conditions is possible. Figure 5a shows the more detailed information on the effect of relative humidity on the proton conductivity through the thin fiber composite and pristine PFSA membranes at 80 °C. Temperature dependence of the proton conductivity through the PFSA thin fiber composite and pristine PFSA membranes at 90% RH is shown in Figure 5b. The composite membranes containing 10 and 15 wt % PFSA thin fibers showed higher proton conductivity in the wide range of temperature. The activation energy (*E*_a_) of the proton conductivity through the thin fiber composite membranes (7.7–9.8 kJ mol^–1^) compares with that through the pristine Aquivion membranes (8.5 kJ mol^–1^) (these values also compare with the reported *E*_a_ value for the short-side-chain PFSA of 6.7 kJ mol^–1^^31^), indicating intrinsic superior proton conduction of the solid-state Aquivion with and without fiber framework. On the contrary, the composite membrane containing 15 wt % ion-insulating PVDF thin fibers showed the lower proton conductivity and higher *E*_a_ value of 22 kJ mol^–1^ more than expected (the temperature dependence of the proton conductivity is shown in Figure S8). These findings clearly indicate that a certain amount of ion (proton)-conductive thin fiber webs sufficient to form the well-interconnected fiber framework in the membranes substantially contributes to the proton transport through the membranes.^14^ One possibility is that the orientation of PTFE crystallites described above may support the connection of ionic domains or amorphous domains inside the fibers. In the presence of water (i.e., high humidity condition), swelling of the ionic domains would improve their connection, resulting in the formation of efficient proton transport pathways inside both the Aquivion matrix and thin fibers in the composite membranes.

**Table 1:** Physicochemical Properties of the Thin Fiber Composite and Pristine PFSA Membranes

membrane	IEC [mmol g^–1^]	water content [%]	proton conductivity [mS/cm]	
			@ 80 °C, 40% RH	@ 80 °C, 90% RH
PFSA composite (5 wt % thin fiber)	1.3	35	4.3	8.3
PFSA composite (10 wt % thin fiber)	1.3	36	5.0	8.7
PFSA composite (15 wt % thin fiber)	1.3	35	6.2	8.6
pristine PFSA	1.3	36	4.0	8.2
PVDF composite (15 wt % thin fiber)	1.0	27		2.9

The additive effects of thin fibers on the mechanical properties (i.e., strength, Young’s modulus, tensile strength, and elongation at break) of the prepared composite membranes were obtained from the stress–strain (S–S) curves (typical S–S curves are shown in Figure S7). These results are summarized in Table 2. All the prepared PFSA thin fiber composite and pristine PFSA membranes showed almost same Young’s modulus of 129–136 MPa, and the tensile strength of the thin fiber composite membranes slightly increased with an increase in the content of thin fibers compared to that of the membrane without thin fibers (up to 10%). The membrane elongation, on the other hand, increased by 15–30% by addition of thin fibers in the polymer matrix. These findings corroborate that thin fibers and a matrix are well integrated in the composites. On the other hand, the mechanical properties of the prepared composite membranes were still lower than those of the composite membrane containing the hot-pressed PVDF thin fiber webs (Table 2, typical S–S curves are shown in Figure S9). Thus, further improvement of the mechanical properties will be required for practical application. The thermal properties of the prepared membranes and corresponding discussion^32,33^ are included in the Supporting Information (Figures S10).

**Table 2:** Effect of a Thin Fiber Framework on the Mechanical Properties of the Composite PFSA Membranes

membrane	Young’s modulus [MPa]	tensile strength [MPa]	elongation at break [%]
PFSA composite (5 wt % thin fiber)	132 ± 3	12.7 ± 0.2	122 ± 6
PFSA composite (10 wt % thin fiber)	129 ± 3	13.2 ± 0.7	131 ± 5
PFSA composite (15 wt % thin fiber)	136 ± 9	13.9 ± 1.0	140 ± 10
pristine PFSA	135 ± 9	12.6 ± 0.1	107 ± 15
PVDF composite (15 wt % thin fiber)	297	22.4	165

### Fuel Cell Performance

**Figure 6:**
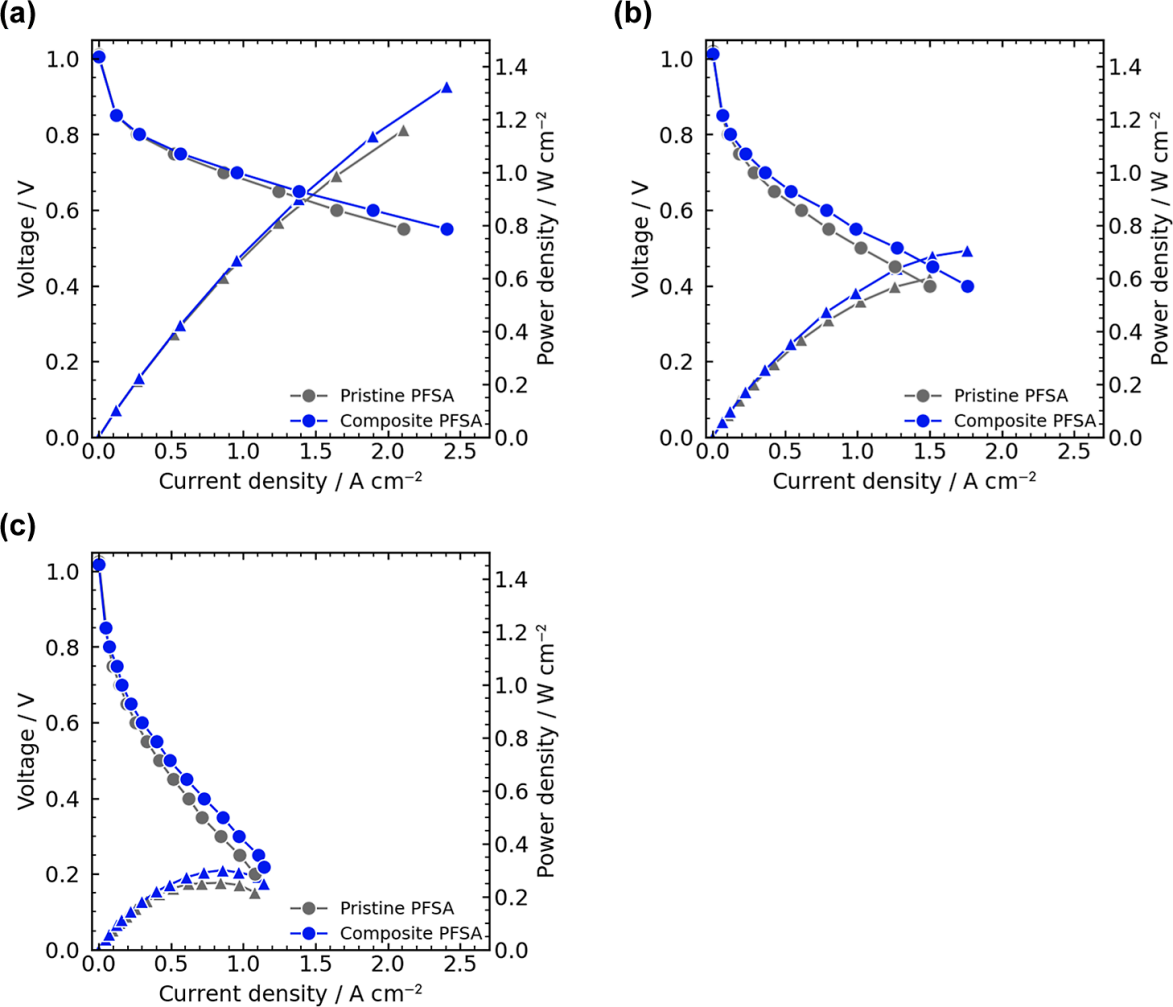
Polarization curves of fuel cells using pristine and composite PFSA membranes (thin fiber content of 15 wt %) at (a) 80 °C (100% RH@anode and 81% RH@cathode), (b) 100 °C (47% RH@anode and 38% RH@cathode), and (c) 120 °C (24% RH@anode and 19% RH@cathode). Circle and triangle represent voltage and power density, respectively.

We evaluated the PEFC performance of the PFSA composite PEMs membranes in a single cell FC using a MEA with an area of 1 cm^2^.^34−39^ Herein, the PFSA composite membrane (15 wt % thin fiber, 39 μm thick) with the highest proton conductivity and mechanical properties and pristine PFSA membrane (36 μm thick) were used. Figure 6 shows the polarization curves of FCs using the PFSA composite and pristine PFSA membranes under three conditions. All cells showed similar open circuit voltages at 1.01–1.03 V, suggesting good gas barrier property of the membranes. The PFSA thin fiber composite membrane outperformed the pristine PFSA one over the entire current density range for all the temperature conditions: the Ohmic polarization of the PFSA thin fiber composite membrane was improved compared with the pristine PFSA one. The obtained peak power density (*P*_max_) values for the PFSA thin fiber composite membrane (1324, 704, and 300 mW cm^–2^ at 80, 100, and 120 °C, respectively) were better than those of the pristine PFSA membrane (1161, 600, and 253 mW cm^–2^ at 80, 100, and 120 °C, respectively); and compared with the previously reported PEFC performances,^15,29,40−44^ particularly for the high-temperature low-humidity condition at 120 °C and ∼25% RH (Table S1).

The Nyquist plots obtained from EIS measurements during FC operation at 0.6, 0.65, and 0.7 V are shown in Figures S11–S13. The Ohmic resistance (*R*_ohm_) was estimated by high-frequency intercept of the impedance arc on the real axis of the Nyquist plots.^45^ Herein, we adopted the data at 0.7 V for discussion because the estimated *R*_ohm_ values were similar for all the operation voltages. At 0.7 V, the *R*_ohm_ values for the PFSA thin fiber composite membrane (40, 120, and 270 mΩ at 80, 100, and 120 °C, respectively) were lower than those for the pristine PFSA membrane (55, 140, and 290 mΩ at 80, 100, and 120 °C, respectively). This trend is consistent with the proton conductivity through the membranes described above (Figure 5). To discuss the effect of PFSA thin fiber webs (i.e., interconnected thin fiber framework) in the composite membranes in detail, we estimated the proton conductivity of the thin fiber webs (σ_fiber_) in the composite membranes from the *R*_ohm_ values obtained from EIS measurements using a first-approximated parallel model as follows^15^
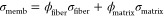
5where σ_memb_, ϕ_fiber_, σ_matrix_, and ϕ_matrix_ represent the proton conductivity of the composite membrane, the volume fraction of thin fiber web (15 vol %, we assume that the volume fraction equals to the weight one of thin fiber web because all components of the composite membrane is Aquivion), proton conductivity of the matrix (we assume that it equals to that of the pristine PFSA membrane), and volume fraction of matrix (85 vol %). The estimated proton conductivity of PFSA thin fiber webs in the composite membranes was higher than those of the matrix, particularly during FC operation (Table 3), indicating the substantial effect of PFSA thin fiber webs on the proton conductivity during FC operation. We think that increasing the volume fraction of PFSA thin fiber webs should be one promising direction for improvement in the proton conductivity through the composite membranes.

**Table 3:** Estimated Proton Conductivity of the PFSA Matrix and Thin Fiber Webs in the Composite Membrane (Thin Fiber Content of 15 wt %) from EIS Measurements Using Eq 5

measurement condition	σ_memb_ [mS/cm]	σ_matrix_ [mS/cm]	σ_fiber_ [mS/cm]
single-cell FC, 80 °C, 100% RH@anode, 0.7 V	96	66	270
single-cell FC, 100 °C, 47% RH@anode, 0.7 V	33	25	79
single-cell FC, 120 °C, 24% RH@anode, 0.7 V	14	12	27
Pt|membrane|Pt cell, 80%, 95% RH	8.6	8.2	11

## Conclusions

In this study, we demonstrated the effects of the PFSA thin fiber framework on the electrochemical and mechanical properties and PEFC performances of all the PFSA thin fiber composite electrolyte membranes: the hot-pressed PFSA thin fiber webs functioned as the well-interconnected fiber framework in the membranes, subsequently enhancing the proton transport and slightly improving the mechanical properties. In addition, the 15 wt % PFSA thin fiber composite PEM showed better FC performance than that of the pristine PFSA one for both the low-temperature high-humidity and high-temperature low-humidity conditions (the *P*_max_ values were improved by 14–19%). To the best of our knowledge, this is the first report on the all PFSA composite membranes containing a PFSA thin fiber framework. We believe that the preparation method of the PFSA thin fiber composite membranes presented here is easily scalable (blow spinning is a promising a high-throughput thin fiber production process^17^) and the all PFSA thin fiber composite electrolyte membranes can be applied to other electrochemical devices such as batteries and water electrolysis as well as PEFCs. At present, we have not accomplished optimization of the PFSA thin fiber composite PEMs. It is expected that the improved proton conductivity and mechanical properties can be obtained by rational tuning of the some structural parameters such as the increased volume fraction of PFSA thin fiber webs, and the increased orientation of PTFE crystallites and crystallinity of PTFE domain inside thinner fiber,^46^ and thinner PEMs less than 10 μm (target thickness in fuel cell roadmap by NEDO:^47^ 8 μm in 2030). Currently further studies are in progress, and the results will be reported.

## Supporting Information Available

Schematic of apparatus used for blow spinning; surface SEM image and the fiber diameter distributions, determined by SEM image analysis, for the hot-pressed PVDF thin-fiber webs; surface SEM images and their fiber diameter distributions determined by SEM image analysis for the as-spun aligned PFSA thin fiber sheets with a second-order parameter *S* of 0.86; typical TEM image of the post-treated PFSA thin fiber; FTIR spectra of the all PFSA thin fiber composite PEMs after acid treatment; surface SEM image and the fiber diameter distributions, determined by SEM image analysis, for the hot-treated PFSA thin-fiber webs after acid treatment; typical stress–strain curves of the pristine and composite PFSA membranes; temperature dependence of the proton conductivity through the PVDF thin-fiber composite and PFSA thin-fiber composite membranes at 90% RH; stress–strain curve of the PVDF thin-fiber composite and PFSA thin-fiber composite membranes; typical TGA and DSC curves of the pristine and composite PFSA membranes; reported fuel cell performances for the representative PEMs; and Nyquist plots for the fuel cells operated in 0.7, 0.65, and 0.6 V (PDF)
