# Supplementary material for: All-Perfluorosulfonated-Ionomer Composite Membranes Containing Blow-Spun Fibers: Effect of a Thin Fiber Framework on Proton Conductivity and Mechanical Properties
Source: ACS Appl Mater Interfaces. 2024 Feb 21;16(8):10682–91. doi: 10.1021/acsami.3c17643 (PMC10910440; doi:10.1021/acsami.3c17643)
Supplement: Supplementary file 1 — am3c17643_si_001.pdf [file am3c17643_si_001.pdf]

Supporting Information

All-Perfluorosulfonated-Ionomer Composite Membranes Containing Blow-Spun Fibers: Effect of a Thin Fiber Framework on Proton Conductivity and Mechanical Properties

Shuta Onuki,[†, ¶] Yoshiki Kawai,[†,*, ¶] Hiroyasu Masunaga,[‡] Noboru Ohta,[‡] Ryohei Kikuchi,[§] Minoru Ashizawa,[†] Yuta Nabae[†], and Hidetoshi Matsumoto[†,*]

[†] Department of Materials Science and Engineering, Tokyo Institute of Technology, 2-12-1 Ookayama, Meguro-ku, Tokyo 152-8552, Japan

[‡]Japan Synchrotron Radiation Research Institute, 1-1-1 Kouto, Sayo, Hyogo 679-5198, Japan

[§]Materials Analysis Division, Open Facility Center, Tokyo Institute of Technology, 2-12-1 Ookayama, Meguro-ku, Tokyo 152-8550, Japan

*Address correspondence to matsumoto.h.ac@m.titech.ac.jp and kawai.y.ag@m.titech.ac.jp

[¶] Shuta Onuki and Yoshiki Kawai contributed equally.

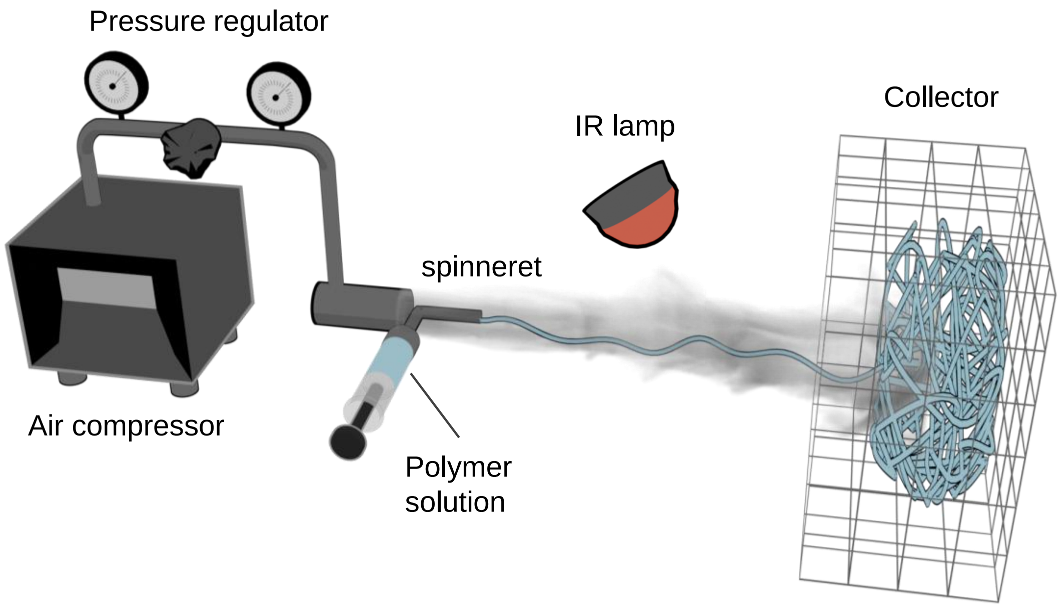

Figure S1. Schematic of the apparatus used for blow spinning.

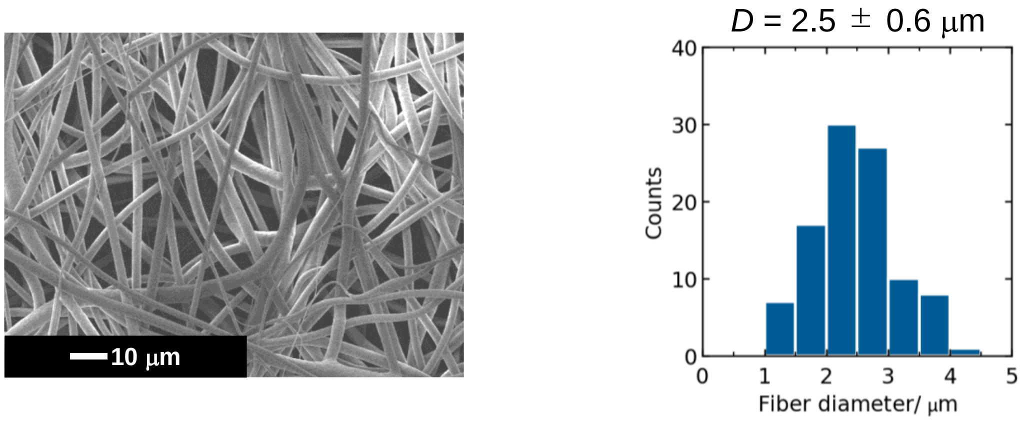

Figure S2. Surface SEM image and the fiber diameter distributions, determined by SEM image analysis, for the hot-pressed PVDF thin-fiber webs.

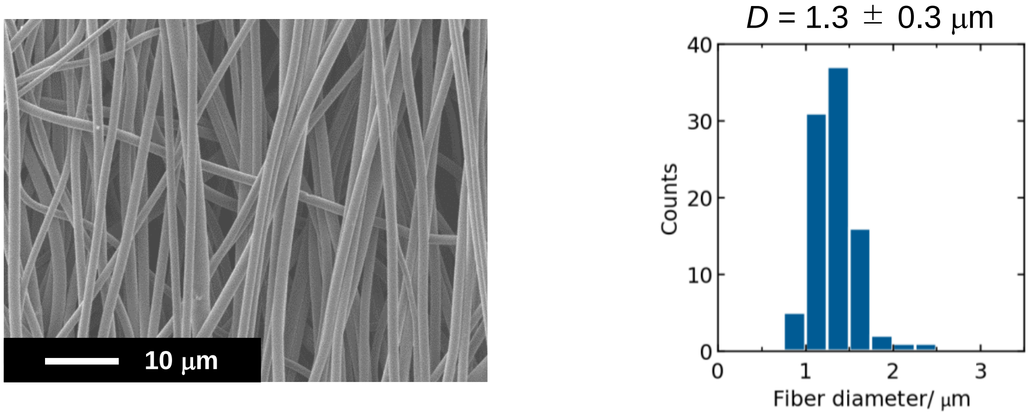

Figure S3. Surface SEM image and the fiber diameter distributions, determined by SEM image analysis, for the as-spun aligned PFSA thin-fiber sheets with a second-order parameter S of 0.86. The alignment of the thin fibers in the sheets was determined from the SEM images using a second-order parameter calculated using the ImageJ software. The second-order parameter S was defined according to the following equation [1]:

$$S = 2(cos^2\theta) - 1 = cos2\theta \qquad (1)$$

where θ is the angle between the direction of the fibers and vertical direction in the SEM image.

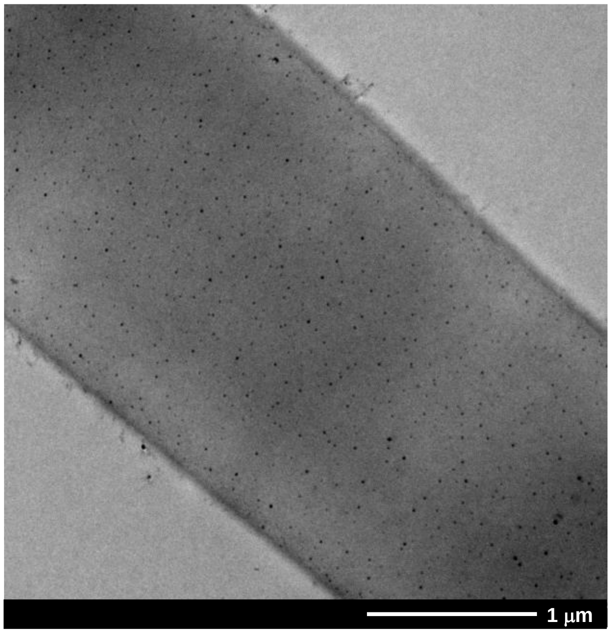

Figure S4. Typical TEM image of the post-treated PFSA thin fiber. The counterions of the PFSA thin fiber were exchanged with Ag^{+}.

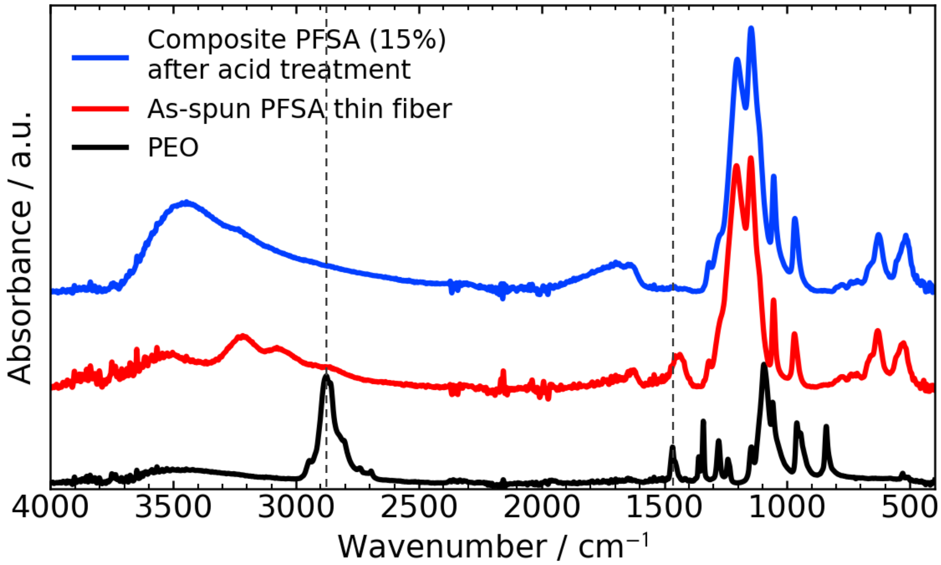

Figure S5. FTIR spectra of the all-PFSA thin-fiber composite PEMs (thin fiber content of 15 wt%) after acid treatment.

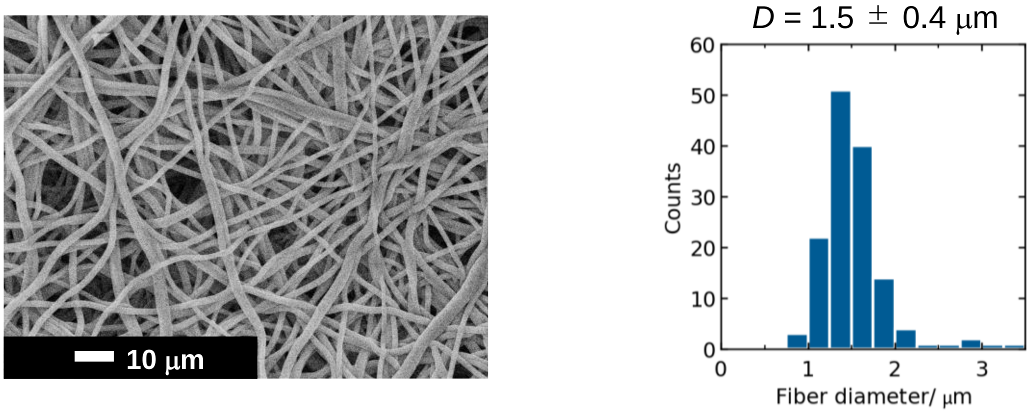

Figure S6. Surface SEM image and the fiber diameter distribution, determined by SEM image analysis, for the hot-treated PFSA thin-fiber webs after acid treatment.

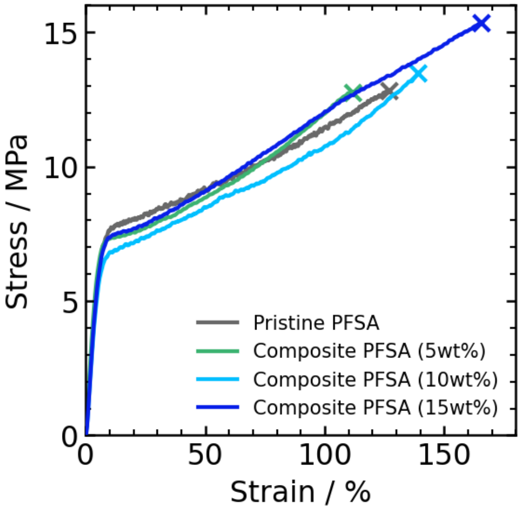

Figure S7. Typical stress–strain curves of the pristine and composite PFSA membranes.

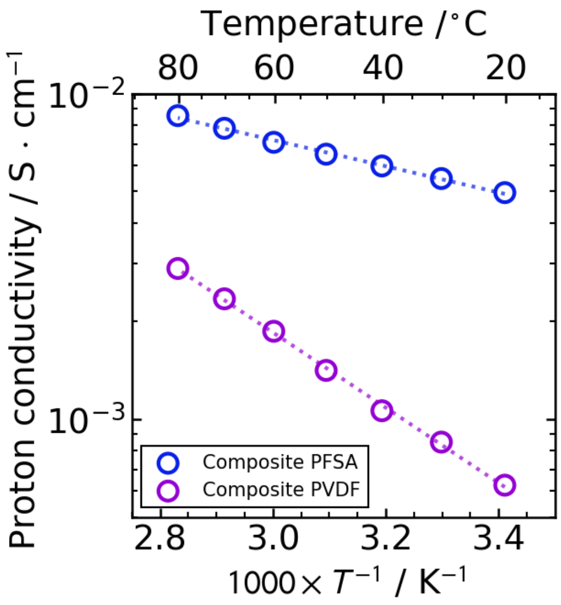

Figure S8. Temperature dependence of the proton conductivity through the PVDF thin-fiber composite (15 wt%) and PFSA thin-fiber composite (15 wt%) membranes at 90 %RH.

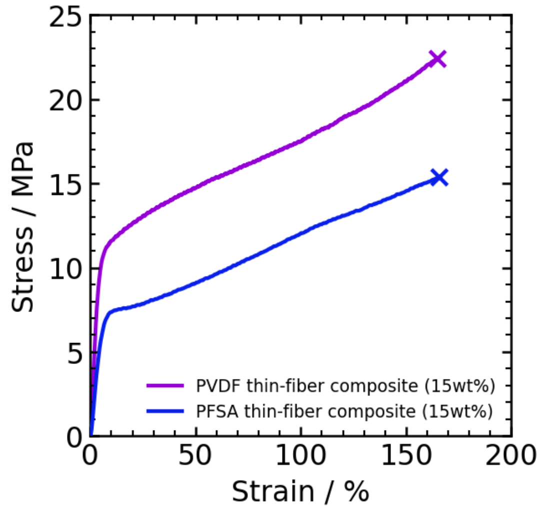

Figure S9. Stress–strain curve of the PVDF thin-fiber composite (15 wt%) and PFSA thin-fiber composite (15 wt%) membranes.

Figure S10 shows typical TGA and DSC curves of the pristine and composite PFSA membranes. Both membranes exhibited a similar thermal behavior. For the TG curves, the gradual weight loss of approximately 6-8 % in the range from 25 to 180 °C would be due to the loss of water molecules. The sharp weight loss at approximately at 280 °C would be associated with desulfonation [2]. For the DSC curves, an endothermic peak near 113 ℃ attributed to the glass transition behavior of amorphous ionic domains of PFSA was observed [2].

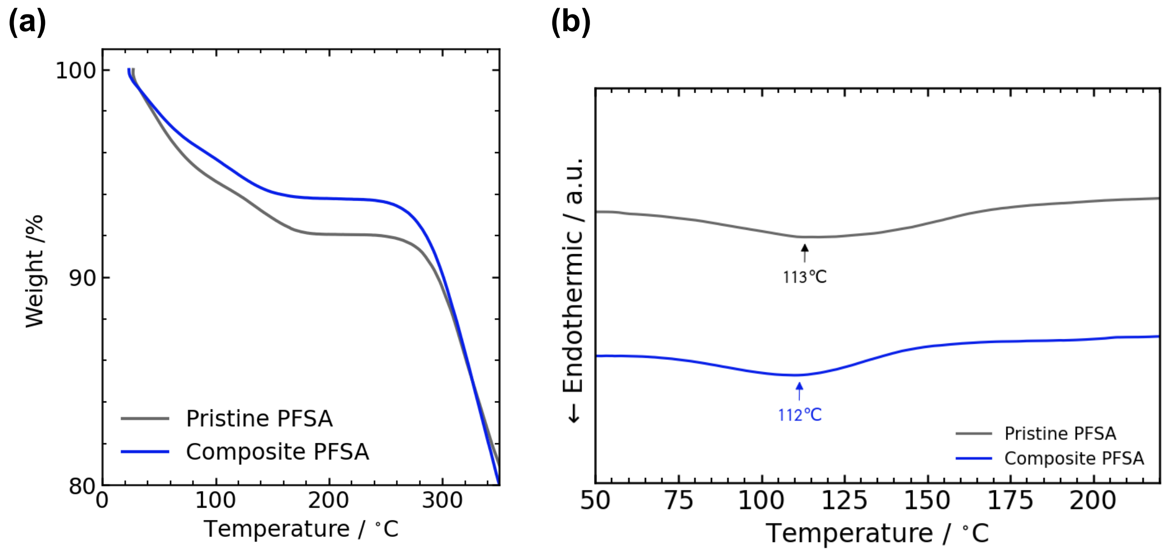

Figure S10. Typical (a) TGA and (b) DSC curves of the pristine and composite PFSA membranes (thin fiber content of 15 wt%).

Table S1. Reported FC performances of representative PEMs

PEM	Thickness [um]	Temperature [°C]	RH [%]	P_{max} [mW cm^{-2}]	Conditions	Reference
**Composite PFSA (15 wt%)**	**39**	**80**	**100**	**1,324 @0.05 V**	MEA area: 1cm^2, 0.4 mg-Pt cm^{-2}, H_2/O_2 without back pressure	**This Work**
	**36**	**120**	**25**	**300 @0.35 V**		
PhySPI: PBI(8:2)BNF /Nafion	5	80	100	680*	@ 0.4V 25cm^2, 0.4 mg-Pt cm^{-2}, H_2/O_2 without back pressure	[3]
		120	24	176		
Nafion NR-211	25	80	100	520*		
		120	24	112		
Nafion polymer	20	80	95	1,469 @2.6 A cm^{-2}	25cm^2, 0.4 mg-Pt cm^{-2}, H_2/air with backpressure 150 kPa and 140 kPa for anode and cathode, respectively.	[4]
		110	25	153 @0.9 A cm^{-2}		
SCC PFSA	20	80	95	1,588 @2.6 A cm^{-2}		
		110	25	279 @0.9 A cm^{-2}		
Nafion-212	51	110	25	121	5 cm^2, 0.5 mg-Pt cm^{-2}, H_2/O_2 without back pressure	[5]
sPSU-SGO	50–55	110	25	183		
sPSU	50–55	110	25	38		
3M 660/PPSU-NF Composite membrane	51	80	93	630*	@0.6 V 5 cm^2, 0.25 mg-Pt cm^{-2} (prior to fuel cell testing, MEAs were briefly soaked in 1M H_2SO_4), H_2/air without back pressure	[6]
		120	25	144*		
Nafion NR-211	25	80	93	479*		
		120	25	60*		
PVDFHFP/Nafion -DMD	12	80	95	1570	4 cm^2, 0.5 mg-Pt cm^{-2}, H_2/O_2 without back pressure	[7]
		120	35	450		
Nafion HP	20	80	95	1330		

		120	35	420		
PEI/Aquivion-SiO_2/HPA	30–50	120	40	660	No information of MEA area, 0.4 mg-Pt cm^{-2}, H_2/O_2 without back pressure	[8]
Aquivion E87-05S	50	120	40	410		
Aquivion E79-03S (Solvicore 6 cells compressed at 15 kg cm^{-2})		80	100	727 @ 0.72 V		
		110	33	401 @ 0.5V		
Aquivion E79-03S (Solvicore 6 cells compressed at 13 kg cm^{-2})		80	100	600 @0.6 V		
		120	25	237 @0.59 V	360 cm^2, 0.6–0.8 mg-Pt cm^{-2}, H_2/air at 1.5 bar.	[9]
Aquivion E79-03S (JMFC 5 cells compressed at 10 kg cm^{-2})	30	80	100	675		
		110	33	370		
Aquivion E79-03S (Solvicore 5 cells compressed at 10 kg cm^{-2})		80	100	730		
		120	25	261		

*Calculated data from Reference.

Abbreviations: PFSA, perfluorosulfonic acid; Phy, phytic acid; SPI, sulfonated polyimide; PBI, polybenzimidazole; BNF, blend polymer nanofibers; SSC, short-side-chain; sPSU, sulfonated polysulfone; SGO, organo-sulfonated derivative of the graphene oxide; 3M 660, Perfluorosulfonic acid with EW of 660 from 3M Company; PPSU-NF, polyphenylsulfone nanofiber; PVDF-HFP, poly(vinylidene fluoride-*co*-hexafluoropropylene; DMD, direct membrane deposition; PEI, poly(ether imide); SiO_2/HPA, SiO_2/heteropolyacid nanoparticles; JMFC, Johnson Matthey fuel cells.

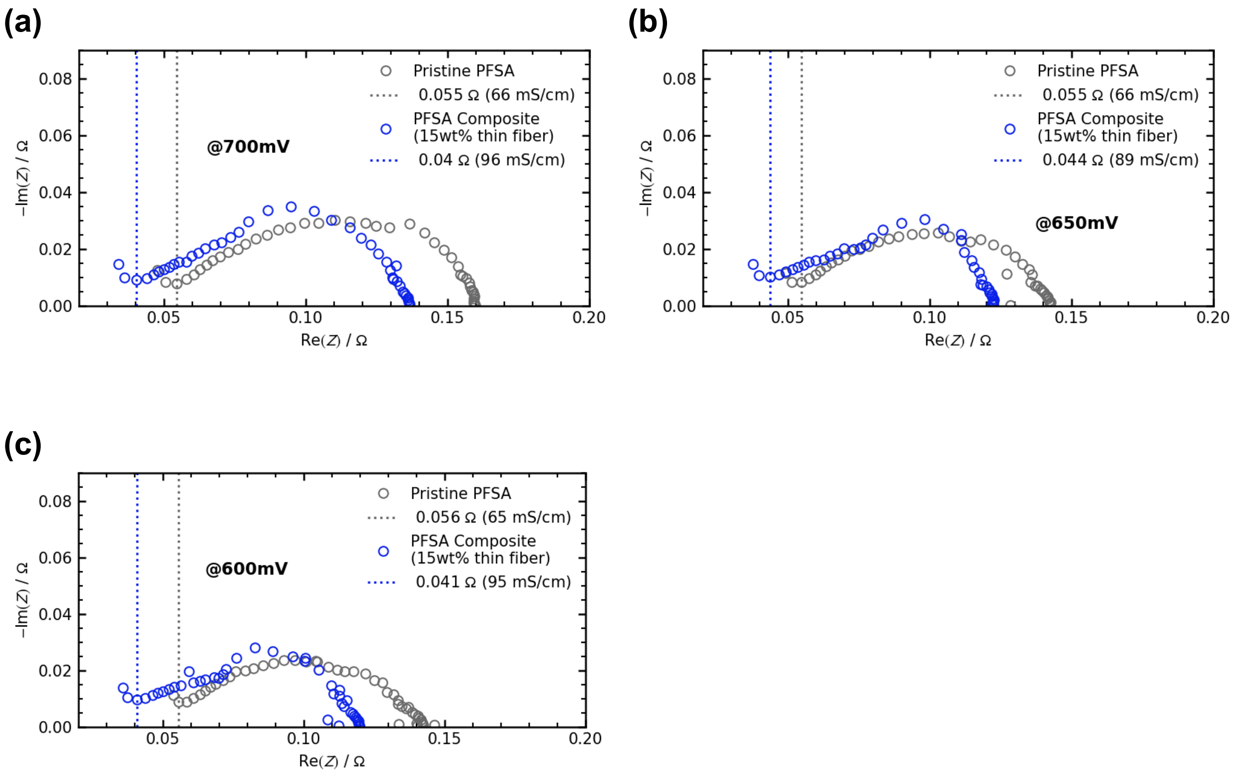

Figure S11. Nyquist plots of the FCs operated in (a) 0.7 V, (b) 0.65 V, and (c) 0.6 V at 80°C (100% RH@anode and 81% RH@cathode). The dotted lines show R_{ohm}.

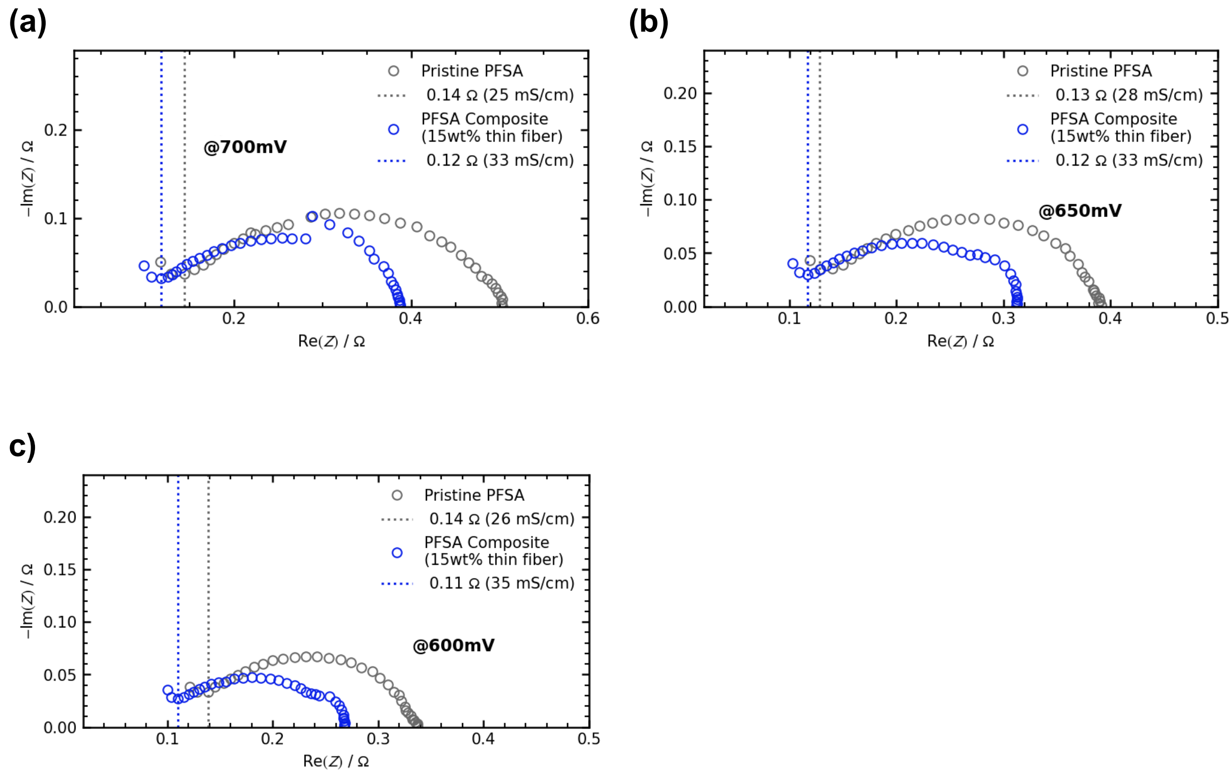

Figure S12. Nyquist plots of the FCs operated in (a) 0.7 V, (b) 0.65 V, and (c) 0.6 V at 100°C (47% RH@anode and 38% RH@cathode). The dotted lines show R_{ohm}.

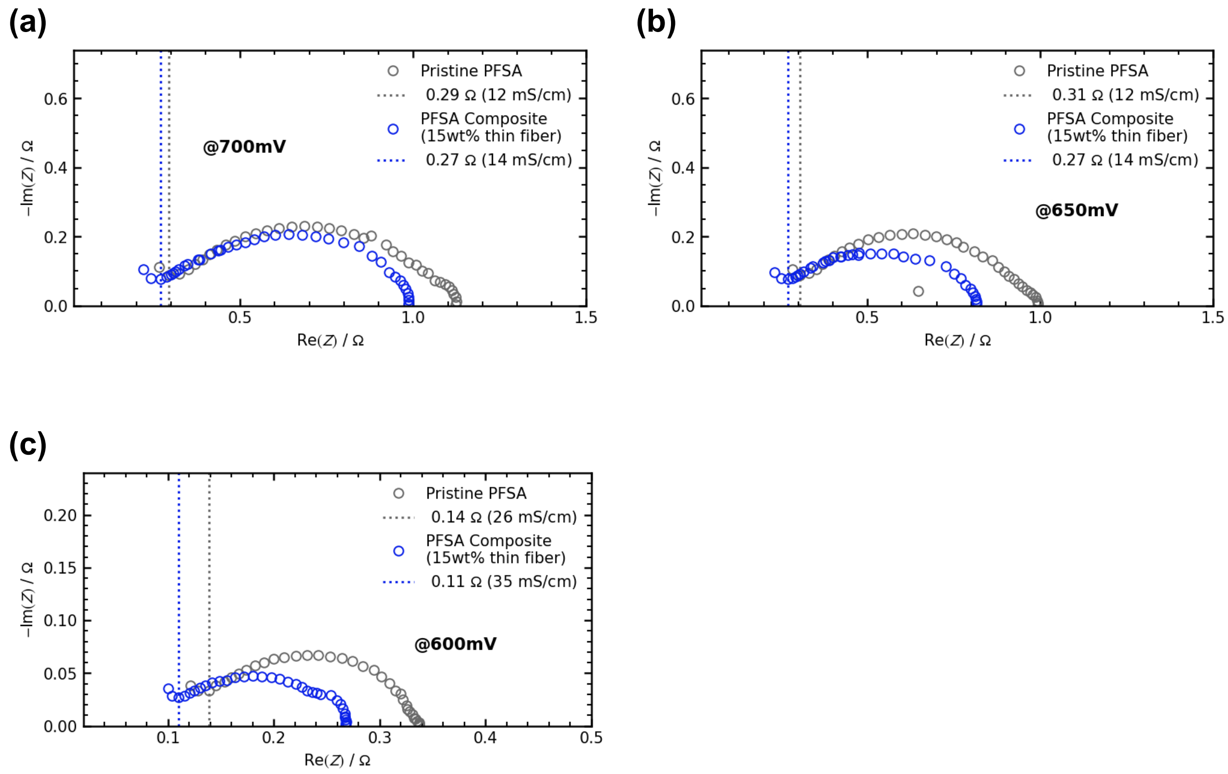

Figure S13. Nyquist plots of the FCs operated in (a) 0.7 V, (b) 0.65 V, and (c) 0.6 V at 120°C (24% RH@anode and 19% RH@cathode). The dotted lines show R_{ohm}.

REFERENCES

1. Li, D.; Takarada, W.; Ashizawa, M.; Yamamoto, T.; Matsumoto, H. Effect of Hydrogen–Deuterium Exchange in Amide Linkages on Properties of Electrospun Polyamide Nanofibers. *Polymer* **2021**, *229*, 123994. DOI: 10.1016/j.polymer.2021.123994.
2. de Almeida; S. H., Kawano, Y. Thermal Behavior of Nafion Membranes. *J. Therm. Anal. Calorim.* **1999**, *58*, 569–597. DOI: 10.1023/A:1010196226309
3. Suzuki, K.; Tanaka, M.; Kuramochi, M.; Yamanouchi, S.; Miyaguchi, N.; Kawakami, H. Development of Blend Nanofiber Composite Polymer Electrolyte Membranes with Dual Proton Conductive Mechanism and High Stability for Next-Generation Fuel Cells. *ACS Appl. Polym. Mater.* **2023** *5*, 5177–5188, DOI: 10.1021/acsapm.3c00656
4. Guan, P.; Zou, Y.; Zhang, M.; Zhong, W.; Xu, J.; Lei, J.; Ding, H.; Feng, W.; Liu, F.; Zhang, Y. High-Temperature Low-Humidity Proton Exchange Membrane with "Stream-Reservoir" Ionic Channels for High-Power-Density Fuel Cells. *Sci. Adv.* **2023**, *9*, eadh1386. DOI: 10.1126/sciadv.adh1386
5. Simari, C.; Lufrano, E.; Brunetti, A.; Barbieri, G.; Nicotera, I. Polysulfone and Organo-Modified Gaphene Oide for New Hybrid Proton Exchange Membranes: A Green Alternative for High-Efficiency PEMFCs. *Electrochim. Acta* **2021**, *380*, 138214. DOI: 10.1016/j.electacta.2021.138214
6. Ballengee, J. B.; Haugen, G. M.; Hamrock, S. J.; Pintauro, P. N. Properties and Fuel Cell Performance of a Nanofiber Composite Membrane with 660 Equivalent Weight Perfluorosulfonic Acid. *J. Electrochem. Soc.* **2013**, *160*, F429. DOI: 10.1149/2.088304jes
7. Breitwieser, M.; Klose, C.; Klingele, M.; Hartmann, A.; Erben, J.; Cho H.; Kerres J.; Zengerle, R.; Thiele, S. Simple fabrication of 12 μm thin nanocomposite fuel cell membranes by direct

electrospinning and printing. *J. Power Sources* **2017**, *337*, 137–144. DOI: 10.1016/j.jpowsour.2016.10.094

8. Lee, C.; Na, H.; Jeon, Y.; Hwang, H. J.; Kim, H.-J.; Isao Mochida, I.; Yoon, S,-H.; Park, J.-I.; Shul, Y.-G. Poly(ether imide) Nanofibrous Web Composite Membrane with SiO_2/Heteropolyacid Ionomer for Durable and High-Temperature Polymer Electrolyte Membrane (PEM) Fuel Cells. *J. Ind. Eng. Chem.* **2019**, *74*, 7–13. DOI: 10.1016/j.jiec.2019.01.034

9. Aricò, A. S.; Di Blasi, A.; Brunaccini, G.; Sergi, F.; Dispenza, G.; Andaloro, L.; Ferraro, M.; Antonucci, V.; Asher, P.; Buche, S.; Fongalland, D.; Hards, G. A.; Sharman, J. D. B.; Bayer, A.; Heinz, G.; Zandonà, N.; Zuber, R.; Gebert, M.; Corasaniti, M.; Ghielmi, A.; Jones, D. J. High Temperature Operation of a Solid Polymer Electrolyte Fuel Cell Stack Based on a New Ionomer Membrane. *Fuel Cells* **2010**, *10*, 1013–1023. DOI: 10.1002/fuce.201000031
